# Dopamine Receptor-Interacting Protein 78 Acts as a Molecular Chaperone for CCR5 Chemokine Receptor Signaling Complex Organization

**DOI:** 10.1371/journal.pone.0040522

**Published:** 2012-07-16

**Authors:** Yi-Qun Kuang, Nicholle Charette, Jennifer Frazer, Patrick J. Holland, Kathleen M. Attwood, Graham Dellaire, Denis J. Dupré

**Affiliations:** 1 Department of Pharmacology, Faculty of Medicine, Dalhousie University, Halifax, Nova Scotia, Canada; 2 Department of Pathology, Biochemistry and Molecular Biology, Faculty of Medicine, Dalhousie University, Halifax, Nova Scotia, Canada; University of Leuven, Rega Institute, Belgium

## Abstract

Chemokine receptors are members of the G protein-coupled receptor (GPCR) family. CCR5 and CXCR4 act as co-receptors for human immunodeficiency virus (HIV) and several efforts have been made to develop ligands to inhibit HIV infection by blocking those receptors. Removal of chemokine receptors from the cell surface using polymorphisms or other means confers some levels of immunity against HIV infection. Up to now, very limited success has been obtained using ligand therapies so we explored potential avenues to regulate chemokine receptor expression at the plasma membrane. We identified a molecular chaperone, DRiP78, that interacts with both CXCR4 and CCR5, but not the heterodimer formed by these receptors. We further characterized the effects of DRiP78 on CCR5 function. We show that the molecular chaperone inhibits CCR5 localization to the plasma membrane. We identified the interaction region on the receptor, the F(x)6LL motif, and show that upon mutation of this motif the chaperone cannot interact with the receptor. We also show that DRiP78 is involved in the assembly of CCR5 chemokine signaling complex as a homodimer, as well as with the Gαi protein. Finally, modulation of DRiP78 levels will affect receptor functions, such as cell migration in cells that endogenously express CCR5. Our results demonstrate that modulation of the functions of a chaperone can affect signal transduction at the cell surface.

## Introduction

Chemokine receptors are a specialized subset of the superfamily of seven transmembrane proteins, coupled to the heterotrimeric G protein. Among the chemokine receptors, CXCR4 and CCR5 have been the subject of many studies demonstrating their important role as co-receptors for M and T-tropic HIV infections, and their involvement in different diseases including cancer and inflammation [1], [2]. While we know very well that G protein coupled receptors (GPCR) signal via multiple proteins assembled into a complex, chemokine receptors are left largely uncharacterized in terms of their association with signaling partners and anterograde trafficking to the plasma membrane. Although oligomerization of GPCRs has been shown for several receptors including CCR5 and CXCR4 [3], [4], [5], [6], [7], very little is known about the factors or proteins that will influence receptor oligomerization, and how specificity of signalling complex organization is attained. Oligomerization of GPCRs can profoundly modify the pharmacology of interacting partners. Allosteric modulation of ligand binding, alteration in G protein signaling and coupling are all associated with GPCR oligomerization [8]. Receptor oligomerization likely occurs via a defined sequence of events, as is the assembly of the different signalling partners [9], [10], [11]. While screening for interactions between chaperones/scaffold proteins and GPCRs, we observed the interaction of a molecular chaperone, DRiP78, with both chemokine receptors CCR5 and CXCR4.

Dopamine Receptor-interacting Protein 78 (DRiP78;also designated by DNAJC14, Jiv and HDJ3) is a member of the Hsp40 family of chaperone proteins [12]. Proteins in this family contain a 70 amino acid motif, the J-domain, important for the recruitment of HSP70 family members and stimulate ATP hydrolysis during the chaperoning process. The human DNAJC family contains 23 members with the presence of the J-domain as the single common feature. These proteins have been shown to play a role in various biological functions, including mitochondrial import, translation, endocytosis and exocytosis, to name a few [13]. DRiP78, an ER-membrane bound chaperone, has been associated with the regulation of the transport of several GPCRs, including D1 dopamine, M2 muscarinic, AT1 angiotensin type II, adenosine and β2-adrenergic receptors to the plasma membrane [14], [15], [16], [17]. DRiP78 was also shown to be involved in the assembly of the G protein subunits Gβγ [17].

Given the regulation of several components of GPCR signalling complexes by DRiP78, and our observation of the interaction of DRiP78 with chemokine receptors, we were interested in characterizing the effects of DRiP78 on the formation of homo and heterodimeric chemokine receptor signalling complexes. We show that DRiP78 is involved in the assembly of homodimeric receptor complexes, but does not affect the heterodimeric receptor assembly. Using CCR5 as our receptor model, we identified the motif responsible for its interaction with the chaperone DRiP78, and demonstrate the effect of this chaperone on the assembly of the G protein subunits with CCR5. Our study is one of the first addressing the specificity of organization of GPCR signalling complexes. Molecular chaperones like DRiP78 may represent a new target for the regulation of receptor expression levels at plasma membrane, and ultimately, the capacity of those receptors to bind chemokine and viruses like HIV-1 at the cell surface.

## Results

### DRiP78 Interaction with Chemokine Receptors

**Figure 1:**
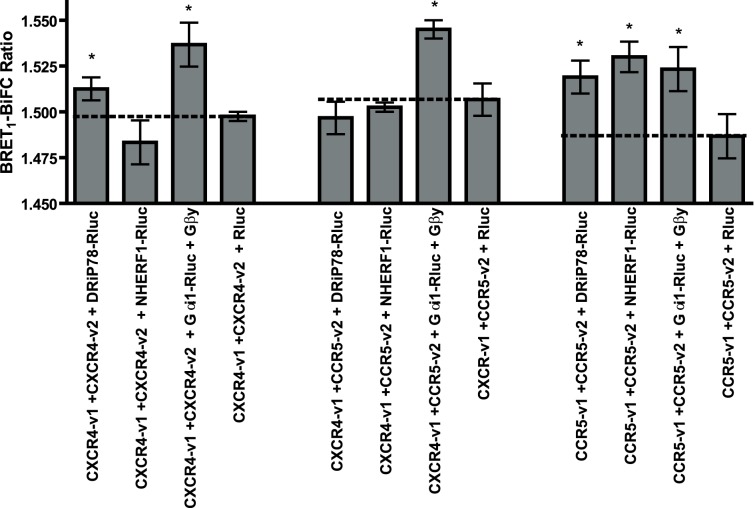
Interaction of DRiP78 with chemokine receptor dimmers. HEK293 cells were co-transfected for 48 hours with various contructs harboring non-functional parts of a YFP variant, venus. Upon interaction between two receptors, the venus parts can associate together and regenerate a functional fluorescent signal. Receptor pairs were expressed with DRiP78-*R*luc and bioluminescence resonance energy transfer was measured. * = *p*<0.05 compared with controls.

Our group and others have previously described that CCR5 and CXCR4 can interact together to form a receptor signalling complex at plasma membrane [3], [18], [19]. Yet, it is not well known how the specific assembly of those complexes is regulated. As previously described [19], we used a fluorescence-based technique, Bimolecular Fluorescence complementation (BiFC) as a tool to obtain a fluorescence signal when two receptors dimerize. The advantage of using this technique is that it allows another fluorescent- or bioluminescent-tagged protein to be used in FRET or BRET, therefore permitting the monitoring of specific interactions of a given combination of receptor pair with other signaling partners. To do so, we used our CXCR4 and CCR5 chemokine receptors tagged with the first 157 amino acids of venus, a yellow fluorescent protein (YFP) variant, or with the 158–238 remaining amino acids of venus. As previously described for BiFC [19], [20], expression of each construct individually does not produce fluorescence, while expression of both parts, when paired to interacting proteins, will generate a functional fluorescent protein. While performing a screen with proteins known to regulate GPCR signaling or trafficking for targets interacting with chemokine receptor dimers, we identified DRiP78 as a potential interactor of CCR5. In Figure 1, we show the BiFC-BRET results of some previously determined *R*luc-tagged positive interactors (NHERF1 and Gαi), DRiP78 or our negative control, *Renilla* luciferase (*R*luc) in presence of CXCR4-v1/v2 homodimer, CCR5-v1/v2 homodimer, or CXCR4-v1/CCR5-v2 heterodimer. Previous results demonstrated that very little fluorescence could be observed with the reversed heterodimer (CCR5-v1/CXCR4-v2) [19], therefore this pairing was not studied here. As previously determined, Gαi interacts with all receptor dimers tested, while NHERF1 could interact only with the CCR5 homodimer. Our results show that DRiP78 can interact with CXCR4 and CCR5 homodimers, but our experiments failed to demonstrate a positive interaction with the heterodimer. Although our BiFC-BRET results suggest some specificity of interaction of DRiP78 with the homodimers, we wanted to confirm those results via co-immunoprecipitation. In order to perform subsequent experiments for the characterization of interacting partners, we used an anti-GFP antibody exhibiting the capacity to recognize only the fully folded and reconstituted form of the fluorescent protein tag, and not the non-fluorescent portions of YFP-variant venus [19]. Having an antibody that recognizes only the reconstituted venus allows the immunoprecipitation of only the receptor dimer we are interested in, and therefore eliminates the background noise that we would obtain with the individual constructs (or v1/v1, v2/v2 dimers). This system allows us to specifically isolate one receptor pair, among a pool of different receptors expressed in a cell.

### CCR5 Domain Responsible for DRiP78 Interaction

**Figure 2:**
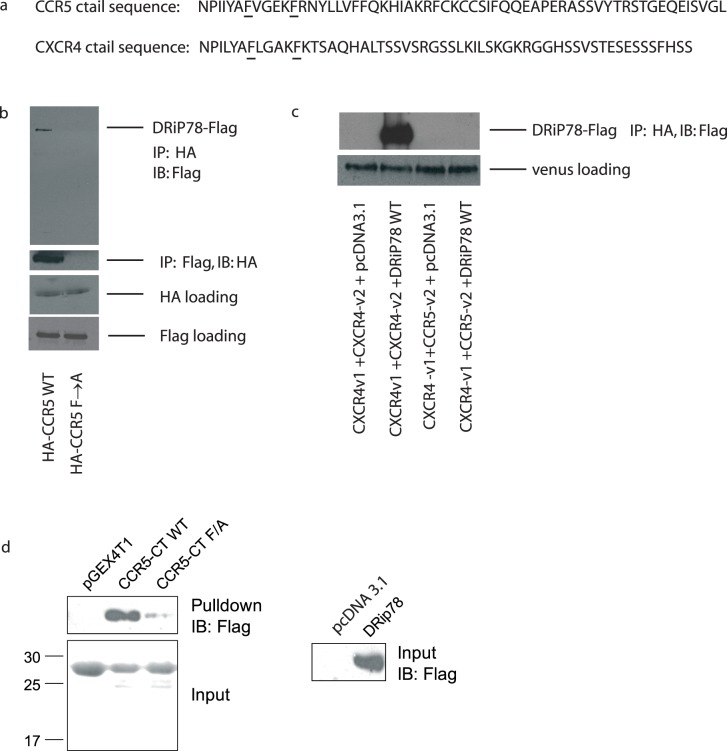
DRiP78 interaction with CCR5 homodimers. a) CXCR4 and CCR5 sequences corresponding to the potential interaction site of DRiP78, starting at the beginning of the carboxy-terminal tail of the receptor. b) Co-immunoprecipitation of DRiP78 with CCR5 WT or with a mutation of the potential interaction site (phenylalanine residues mutated to alanine residues) and with CXCR4-v1/CCR5-v2. c) Interaction of CXCR4 with DRiP78. d) CCR5 c-tail interaction with DRiP78. The GST-CCR5 c-tails (wild type or F/A mutant) were incubated HEK293A cell lysates expressing DRiP78 to detect the interaction.

Both CXCR4 and CCR5 exhibit the predicted interaction site for DRiP78 (underlined in Figure 2a). It was previously suggested that a short region of the proximal C terminus of the D1 receptor serves as an ER-export signal [14]. Substitution of hydrophobic residues within this motif, which are conserved among almost all rhodopsin-like GPCRs [21], results in the receptor being retained in the ER and dysfunctional maturation. DRiP78 was then shown to specifically interact with this motif. It was found that a four-amino-acid spacing of hydrophobic residues within the proximal c-terminal region is highly conserved among GPCRs, as part of an α-helical conformation in which at least one hydrophobic residue in each turn forms a non-polar surface [14]. Mutation of the phenylalanine residues which composed in this sequence resulted in the trapping of D1 receptors in the ER and improper maturation. In the chemokine receptors CXCR4 and CCR5, the FxxxxF sequence is present and mutagenesis of the phenylalanine residues to alanine residues in CCR5 results in a loss of receptor interaction with DRiP78. Figure 2b (top panel) shows the co-immunoprecipitation of FLAG-tagged DRiP78, following HA-tagged CCR5 precipitation, The reciprocal experiment was performed. The two bottom panels show the expression of both constructs, as a loading control. Figure 2c shows that CXCR4 can co-immunoprecipitate Flag-tagged DRiP78, while the heterodimer CXCR4-v1/CCR5-v2 was unable to do so. Positive co-immunoprecipiation results for the CCR5 homodimer are also observed in Figure 2b.

To further confirm the interaction of DRiP78 with CCR5, we used GST-tagged c-tails corresponding to the sequence shown in Figure 2a, either the wild type (WT) or with the underlined phenylalanine residues mutated to alanine residues and incubated the purified receptor c-tails in presence of DRiP78. Again, the interaction between DRiP78 and CCR5 was blocked by the mutation of the FxxxF motif (Figure 2d).

### Effect of DRiP78 on CCR5 Localization

**Figure 3:**
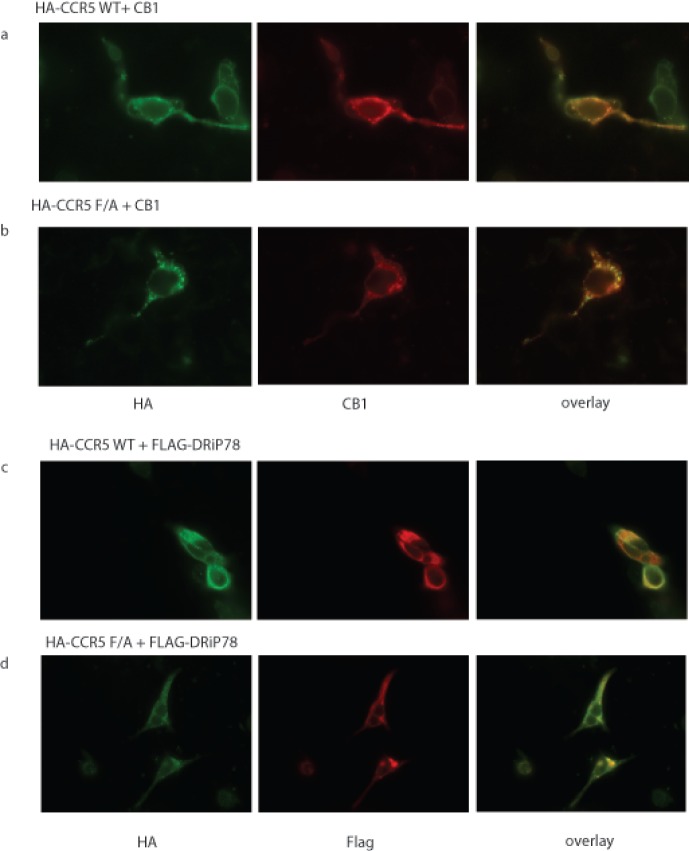
Effect of DRiP78 on CCR5 localization. Cells were plated on coverslips for 24 hours and then transfected with HA-CCR5 (WT (a) or F/A mutant (b)) and a cell surface marker, the CB1 receptor. c) expression of WT CCR5 with DRiP78 and d) Expression of mutated CCR5 with DRiP78. Green shows HA expression, red shows CB1 receptor or FLAG expression while the overlay column shows merged images of the previous two as acquired via fluorescence microscopy. DRiP78 is an ER chaperone, and therefore serves as an ER marker.

As previously mentioned, mutation of the DRiP78 interaction motif resulted in the retention of D1 receptors in the ER, reduced ligand binding, and a slowdown in the kinetics of receptor glycosylation. Here, we evaluated the effect of the FxxxxF change to AxxxxA in the CCR5 c-tail on the cellular localization of the receptor. Figure 3a shows the expression at plasma membrane of HA-tagged WT CCR5, colocalized with a receptor known to localize at the plasma membrane, the cannabinoid CB1 receptor. Conversely, the F/A CCR5 mutant was retained inside the cell (Figure 3b), where it colocalizes with DRiP78 (Figure 3d). Interestingly, overexpression of DRiP78 in the presence of WT CCR5 (Figure 3c) also resulted in a shift of receptor localization from the plasma membrane to ER compartments, where CCR5 colocalizes with DRiP78. DRiP78 is an ER chaperone, and therefore acted as an ER marker in experiments it was used in.

**Figure 4:**
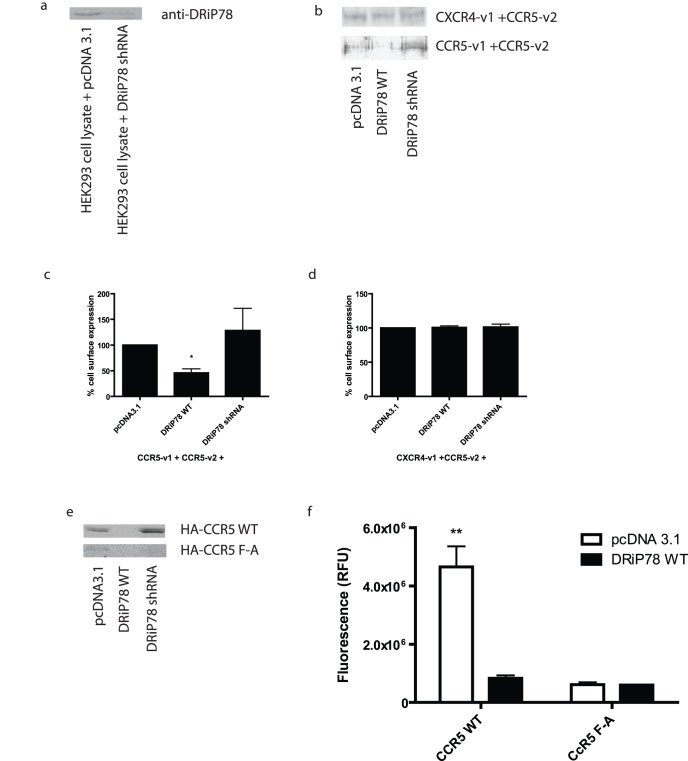
Effect of DRiP78 on Chemokine receptor plasma membrane localization. a) Knockdown levels of DRiP78 expression with the DRiP78-targetting shRNA. 48 hours post-transfection with cDNAs encoding DRiP78 (WT or shRNA) and receptor constructs forming CCR5 homodimers or CXCR4-CCR5 heterodimers, cells were labeled with biotin and precipitated using streptavidin. An immunoblot against GFP was then performed to show receptor levels at plasma membrane. c, and d) Quantification of at least 3 experiments, as performed in b). e) Plasma membrane expression of CCR5 WT or F/A in presence of DRiP78 WT or shRNA. f) Fluorescence measurements of cell surface expression of HA-CCR5 WT and F/A using an ALEXA-488 coupled secondary antibody.

Confirmation of the effect of DRiP78 on plasma membrane localization of CCR5 homodimers was obtained following immunoprecipitation of cell membrane-biotinylated proteins. As observed in Figure 3, DRiP78 overexpression resulted in a decreased localization at plasma membrane for CCR5 (Figure 4b bottom panel, second lane). This effect could be countered by the expression of a DRiP78 shRNA (knockdown shown in Figure 4a), which restored plasma membrane localization of the CCR5 homodimer (Figure 4b, bottom panel, 3^rd^ lane). No effect of DRiP overexpression or knockdown were observed for the CXCR4-v1/CCR5-v2 heterodimer (Figure 4b, top panel), consistent with the absence of interaction of DRiP78 with this complex. The quantification of plasma membrane localization for the CCR5 homodimer, and CXCR4-v1/CCR5-v2 heterodimer is illustrated in Figures 4c and 4d, respectively. Following biotinylation of membrane proteins, we also observed that HA-tagged WT CCR5 could localize to the plasma membrane and its expression was modulated by overexpression (decreased PM expression) or knockdown (increased PM expression) of DRiP78 (Figure 4e, top panel). In comparison, the HA-CCR5 F/A receptor failed to reach the plasma membrane and its PM expression was not modulated by DRiP78 (Figure 4e, bottom panel). Quantification of HA-tagged receptor-associated ALEXA Fluor 488 at plasma membrane shows similar results (Figure 4f).

### Effect of DRiP78 on Receptor Oligomerization

**Figure 5:**
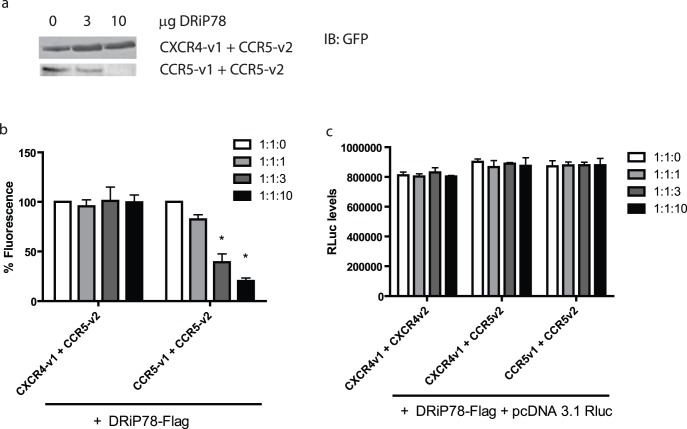
DRiP78 levels in cells affect function. a) Increasing amounts of DRiP78 cDNA were transfected in HEK293 cells and expression levels of chemokine receptor dimers were measured. b) Cells expressing chemokine receptor dimers were transfected for 48 hours with various amounts of cDNA encoding DRiP78 and fluorescence levels were then measured. Fluorescence appears only when receptors dimerize therefore any variation in fluorescence level would be attributed to a change in the capacity of those receptors to associate. c) Quantification of *R*luc levels in cells, when expressed along with constructs mentioned in b). *R*luc levels do not change, so fluorescence levels are not attributed to changes in the cell capacity to produce the proteins.

Dimerization of GPCRs has been previously shown to occur early following biosynthesis, which act as a prerequisite for ER export [7]. Here, we evaluated the effect of increasing levels of DRiP78 overexpressed in HEK293 cells co-expressing various chemokine receptor dimer pairs. Our results show that upon lysis of cells, the levels of CXCR4-v1/CCR5-v2 were not modified, again confirming the absence of an interaction with DRiP78 (Figure 5a). Interestingly, the levels of CCR5 homodimers expressed in the cell were affected, suggesting a role of DRiP78 in the formation of those complexes. Indeed, while overall levels of cDNA transfected remained the same through compensation with empty pcDNA3.1, the levels of homodimers immunoblotted decreased for CCR5. Those results were confirmed via total fluorescence measurements of HEK 293 cells expressing the various ratios of DRiP78 and chemokine receptor pairs, as shown in Figure 5b. Figure 5c shows that if we co-express constant levels of another marker (here, *Renilla* luciferase), its levels are not changed. This suggests that the changes in expression levels of the receptors are not induced by an increase or decrease in cell numbers, otherwise the *R*luc levels would vary.

### Effect of DRiP78 on Receptor Signaling Complex Assembly

**Figure 6:**
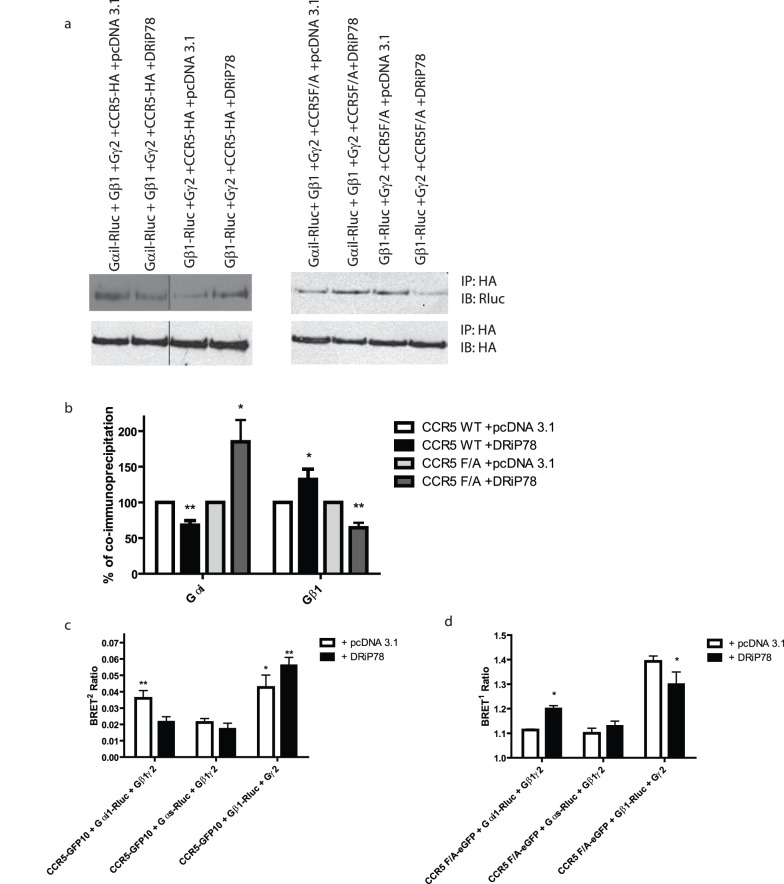
DRiP78 regulates CCR5 interaction with G protein subunits during assembly. a) HEK293 cells were co-transfected with cDNAs encoding DRiP78 WT, HA-CCR5 WT or F/A, and various G protein subunits (Gαi-*R*luc, Gβ1-*R*luc in presence of Gγ2. Cells were then lysed and immunoprecipitations using an antibody directed against HA were performed. An immunoblot was then performed using an antibody directed against *R*luc to reveal the G protein subunits. b) Histogram representation of the results obtained by immunoblotting. c) and d) BRET experiment showing WT CCR5-GFP_10_ or CCR5 F/A-eGFP interaction with *R*luc-tagged Gαi, Gαs and Gβ1 subunits. * = *p*<0.05; ** = *p*<0.01 compared with negative controls. Results are representative of 3 independent experiments.

Several publications from our group and others have demonstrated early assembly of GPCR signaling complexes with their cognate G protein and effector. It was suggested that the Gβγ subunits could interact with the receptor earlier than the Gα subunit, to assemble into a signaling complex [11]. Also, DRiP78 was shown to be involved in the early assembly of Gβγ subunits, along with other chaperones [17]. Since our results suggested an important role of DRiP78 in the assembly of chemokine receptor dimers, we wondered whether DRiP78 could also play a significant role in the assembly of the receptor with the G protein. To study this potential function, we co-expressed HA-CCR5 WT or HA-CCR5 F/A with Gαi-*R*luc, in presence of Gβ1γ2 or with Gβ1-*R*luc + Gγ2. Then, we immunoprecipitated the receptor using the HA epitope and immunoblotted against *R*luc, to reveal the levels of G protein subunits co-precipitated with the receptor. Our results suggest that upon co-expression of DRiP78, the levels of Gαi co-precipitated with the receptor are decreased (Figure 6a, quantified in 6b). Given the decrease in trafficking from the ER towards PM, fewer receptors would get the opportunity to meet with their cognate Gα protein. Conversely, the expression of DRiP78 facilitates the interaction of Gβγ with the receptor. This interaction was shown to occur prior to ER exit for other GPCRs [11]. Therefore, an increase in interaction coincides with a decrease in trafficking from the ER towards PM. Similar results were obtained via BRET, using CCR5-GFP_10_ or CCRF F/A-eGFP, co-expressed with Gαi-*R*luc or Gβγ-*R*luc, in comparison to the negative control Gαs-*R*luc (Figure 6c). A basal interaction between CCR5-GFP_10_ and Gβ1-*R*luc, in presence of Gγ2 is observed, and this interaction is slightly increased upon co-expression of DRiP78. With CCR5 F/A, an increase in the interaction with Gαi is observed in presence of DRiP78, while a decrease of the interaction with Gβ1, in presence of Gγ2 occurs (Figure 6d).

### Effect of DRiP78 on Chemokine Receptor-dependent Migration

**Figure 7:**
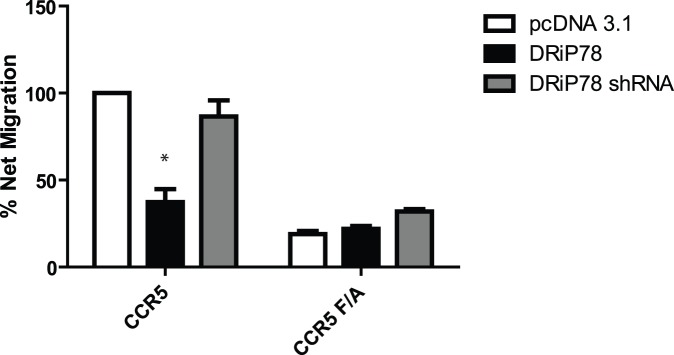
DRiP78 effect on Jurkat cell migration. 48 hours post-transfection, Jurkat cells overexpressing WT CCR5 or CCRF F/A, and DRiP78 constructs were placed in the upper chamber of a transwell plate and incubated with 10 ng/mL of RANTES for 5 hours. Cells that migrated through the membrane were then counted. Results are representative of 3 independent experiments, and * = *p*<0.05 compared with controls.

Signaling from chemokine receptors at the plasma membrane is key to proper functioning of the cell. Our results suggest up to now that DRiP78 is important for the proper assembly of chemokine receptor signaling complexes. We wanted here to evaluate the effect of DRiP78 on chemokine receptors signaling coming from the plasma membrane. To do so, we evaluated the migration potential of Jurkat cells, expressing chemokine receptor CCR5. As described in the methods section, the cells were placed in the upper chamber of a transwell plate, while 10 ng/mL RANTES was present in the bottom chamber. The number of cells passing through the membrane was counted and compared to cells that were not put in presence of the chemoattractant. As predicted from our cell surface expression experiment for CCR5, migration levels are reduced with DRiP78 overexpression for the WT receptor, while the DRiP78 shRNA restored migration to basal levels (Figure 7). Very little migration was observed when the CCR5 F/A mutant receptor was overexpressed, indicative of its intracellular localization. Our results suggest that we can indeed modulate receptor expression by regulating the ER export of a GPCR.

## Discussion

Although GPCRs have been thoroughly characterized in terms of their signaling pathways and drug interactions, the advent of the concept of homo and heterooligomerization has generated a new level of complexity in our understanding of how GPCRs really work. It is likely that drug side effects might be transduced, to a certain level, by receptor heterooligomers, which would then activate signaling pathways we wouldn’t expect. Studying how GPCR oligomers assemble with their signaling partners and traffic to the plasma membrane will certainly aid in understanding the specificity of signal transduction. Inefficient targeting of GPCR oligomers *in vivo* has been shown to be the cause of some pathophysiological disease states, emphasizing the importance of proper receptor assembly and trafficking towards plasma membrane [22], [23]. This study is among the first in attempting to understand how oligomerization of GPCRs is performed and/or regulated. Our work demonstrates that molecular chaperones will display levels of selectivity towards some receptor complexes, and that some chaperones will be involved in the formation of receptor oligomers. Also, the association of oligomers with their cognate signaling partners, such as G proteins may also very well be regulated by molecular chaperones.

Our screen for molecular interactors of chemokine receptors has lead us to the identification of DRiP78, a member of the HSP40 protein family [24]. Up to now, several GPCRs, including the D1-dopamine, M2-Muscarinic, A1-Adenosine, D2-dopamine and β2-adrenergic receptors have been shown to have the ability to interact with DRiP78, and in most cases, can be retained by the chaperone in ER membranes [14], [16], [17]. Those receptors share the common reported DRiP78-recognition sequence [F/L(x)_3,4_FxxxF] within the receptor proximal c-tail. The chemokine receptors CXCR4 and CCR5 share part of this sequence, which appears to be sufficient for DRiP78 to interact. Indeed, CXCR4 and CCR5 do not possess the last phenylalanine residue shown in the recognition sequence, and rather possess solely the F(x)_4_F portion. It was suggested that DRiP78 could monitor and distinguish improperly versus correctly folded proteins, or at least the proper conformation of the receptor c-tail. Small regions of the c-tail could therefore act as conformational switches, which upon masking or revealing would allow proper exit from the ER. As demonstrate by our study, DRiP78 can regulate receptor expression to the plasma membrane of WT receptors. DRiP78 could likely interact with CCR5 and mask the ER exit motif, therefore retaining the receptors inside the ER. The reciprocal also exists, as exemplified by the association of GABA_B_R1 with GABA_B_R2, which allows the heterodimeric complex to be released from the ER and reach the plasma membrane [25]. Therefore, DRiP78 could likely act as a quality control checkpoint, assuring that receptors are properly folded and most likely assembled into competent oligomers with their signaling partners. Other chaperones, such as RTP4, were shown to promote cell surface expression, rather than ER retention and trafficking towards cell surface [26]. RTP4 also appeared to be involved in the specificity of signaling complexes assembled, as it regulated the proportion of μ-δ heterodimers, and thus representing a critical factor influencing the signal transduction induced by exogenous and endogenous ligands.

In terms of specificity, the bovine analog of DRiP78, Jiv, is essential for the polyprotein cleavage and replication of the pestivirus BVDB [27]. Therefore, a role in the maturation of mammalian proteins like GPCRs could be possible. Our study suggests that DRiP78 participates in the formation of receptor dimers, and contributes to the assembly of the G protein with the receptor into a complex. Previous studies demonstrated the interaction of Gβγ with the receptor in the ER, while the interaction with the Gα subunit occurred outside of the ER, but before reaching the Golgi. Our results are in agreement with those from that study, since we show that DRiP78 alters the interaction between CCR5 and Gα. The incapacity of the receptor to leave the ER could explain this result. Whether the regulation of the assembly happens by a direct modulation signaling assembly or by favoring the interaction through retention in the same compartment remains to be determined.

An increasing number of studies have shown that maturation and/or targeting of receptor oligomers to the cell surface are associated with human diseases. Chemokine receptors such as CCR5 demonstrate polymorphisms that induce significant changes in signaling capacities. For example, the CCR5Δ32 mutation retains the receptor intracellularly and confers significant levels of resistance to HIV infection [28]. Retaining the CCR5 receptor has beneficial effects in the prevention of HIV infection. A better knowledge of the molecular mechanisms controlling receptor assembly could help understand how misfolded, polymorphic or mistrafficked receptors influence receptor complex cell surface expression and signal transduction.

## Materials and Methods

### Reagents

Reagents were obtained from the following sources: fetal bovine serum, ALEXA-FLUOR 488 IgG and ALEXA-FLUOR 647 phalloidin and Lipofectamine 2000 transfection reagent were from Invitrogen (Etobicoke, ON, Canada); Dulbecco’s Modified Eagle’s Medium High Glucose, RPMI 1640 Medium, monoclonal anti-FLAG antibody, protein A-Sepharose, crystal violet, forskolin and all chemicals, unless otherwise noted, were from Sigma-Aldrich (Oakville, ON, Canada). EZ-Link Sulfo-NHS-LC-Biotin and Streptavidin Agarose Resin were purchased from Thermo Scientific (Rockford, IL). Polyclonal GFP was purchased from Santa Cruz (Santa Cruz, CA, USA). Coelenterazine H, Covance monoclonal anti-HA raw ascites, Millipore anti-*Renilla* luciferase, Human recombinant RANTES were from Cedarlane Labs, (Hornby, ON, Canada).

### Constructs

CCR5 and CXCR4 receptors constructs were used as previously reported [19]. The CCR5 F/A receptor was subcloned from a pcDNA3.1 vector into pEGFP-N1 using NheI and BamHI. CCR5 was amplified from pcDNA3.1 using the primers: forward:5′-GCAATGGCTAGCACCATGGATTATCAAGTGTCAAGTCCA-3′ and reverse: 5′-GACCTGGGATCCAAGCCCACAGATATTTCCTGCTC-3′ which contained a NheI and BamHI site respectively. PCR was performed by heating the reaction to 95°C for 5 min followed by 30 cycles of 95°C for 30 sec, 55°C for 30 sec and 72°C for 45 sec with a final extension of 72°C for 10 min. The amplified CCR5 sequence was then ligated into a pEGFP-N1 vector that had been cut with NheI and BamHI. All other constructs, including DRiP78 constructs (previously described in [17]) were obtained from Dr. Terence E. Hébert, McGill University, Canada. CB1-CFP was obtained from Melanie Kelly, Dalhousie University.

### Cell Culture and Transfection

HEK293A cells (obtained from Invitrogen, Burlington, ON, Canada) were grown in Dulbecco’s modified Eagle’s medium high glucose supplemented with 10% fetal bovine serum and transfected using Lipofectamine 2000 as per the manufacturer’s instructions. ATCC’s Jurkat cell line TIB-152 (obtained from Cedarlane Labs (Hornby, ON, Canada)) were grown in RPMI 1640. Cells were plated in 6-well plates. Experiments were carried out 48 hours after transfection.

### Bioluminescence Resonance Energy Transfer (BRET) and Bimolecular Fluorescence Complementation (BiFC) Experiments

HEK293 cells were co-transfected with vectors expressing the GFP- and *R*luc-fusion proteins (1 µg of each cDNA was transfected into each well of a 6-well plate, and total DNA/dish was kept constant by adding pcDNA 3.1 vector as required). 48 hours after transfection, cells were harvested and washed once with phosphate-buffered saline (PBS). The cells were then suspended in PBS+ (PBS +0.1% glucose) and distributed into 96-well microplates (white Optiplate; Perkin-Elmer Life and Analytical Sciences). Depending on the GFP variant (GFP10, or eGFP) tagged to our receptors, BRET 1 or BRET2 was selected to perform the experiments. BRET ratios were measured similarly, only the substrate and wavelengths used were slightly different. Signals were collected using coelenterazine H as a substrate for BRET1 at a final concentration of 5 µM. Whether or not BRET occurred was determined by calculating the ratio of the light passed by the 450/58 (luciferase) and 535/25-nm band pass filters (YFP) for BRET1. This ratio is referred to as the BRET ratio. For BRET2, coelenterazine 400a at a final concentration of 5 µM was used. Signals were collected using either 410/80- (luciferase) and 515/30-nm (GFP) band pass filters for GFP10 constructs. To avoid possible variations in the BRET signal resulting from fluctuation in the relative expression levels of the energy donor and acceptor, we designed transfection conditions to maintain constant GFP/*R*luc expression ratios in each experimental set. BiFC signals were determined by the measurement of the light that passed by the 535/25-nm band pass filters (YFP). BRET background was determined under conditions where resonance energy transfer between *R*luc and GFP either could not or did not occur. This was accomplished by expressing *R*luc or *R*luc-tagged proteins either alone or together with GFP or GFP-tagged proteins, none of which interact physiologically. The background was the same regardless of which of the aforementioned individual proteins or combinations of proteins were expressed, and it has been subtracted to yield net BRET.

### Cell Lysis and Immunoprecipitation

48 hours after transfection into 6-well plates, cells were washed with PBS and harvested. Samples were lysed in 0.8 ml of radioimmune precipitation assay buffer (50 mM Tris, pH 7.5, 10 mM MgCl2, 150 mM NaCl, 0.5% sodium deoxycholate, 1% Nonidet P-40, 0.1% SDS, Complete Protease inhibitors (Roche; Laval, QC, Canada) and DNase I). The lysate was solubilized by incubation at 4°C for 30 min, precleared with 50 µl of protein A-Sepharose beads at 4°C for 1 h, and clarified by centrifugation at 14,000 rpm for 10 min. Supernatants were then transferred into another microcentrifuge tube and incubated with an antibody overnight. The immunoprecipitated proteins were eluted from beads with 50 µl of SDS sample buffer and resolved by SDS-PAGE, and Western blots were performed as previously described [29]. When immunoprecipitation was not required, cells were lysed in 200 µl of RIPA buffer, precleared with protein A-sepharose and then SDS-PAGE loading buffer was added. Pulldowns were performed as follows: the wild type or F/A mutant CCR5 tail were cloned into the pGEX4T1 vector. GST fused proteins were then incubated with HEK293 cell lysates overexpressing DRiP78. The incubated beads were washed three times with ice-cold PBS. Beads were then resuspended with 20 µl Laemmli Buffer and boiled 2 min, then loaded on 12% SDS-PAGE gels to detect fusion protein expression.

### Fluorescence Microscopy

Twenty four hours post-transfection, HEK 293 cells were harvested and seeded on laminin-coated coverslips for 4 h at 37°C. The cells were then fixed for 20 min in PBS, pH 7.4, containing 3% (w/v) paraformaldehyde. The coverslips were washed with PBS, drained, and mounted onto glass slides using a drop of 0.4% 1,4-diazabicyclo{2.2.2}octane/glycerol medium. Coverslips were fixed to the slides with nail polish. Fluorescence microscopy was performed with an Olympus IX81 equipped with a Photometrics coolSNAP HQ2 camera and excite series 120Q light source. YFP (venus) was excited at 488 nm, and image acquisition was done at fluorescence emission 525 nm. Alexa-Fluor 647 was also used to label cells.

### Cell Surface-associated Fluorescence

HEK293 cells were co-transfected with the receptor constructs and DRiP78 constructs for 48 hours. After 3 washes with TBS, cells were incubated for 45 minutes on ice in TBS +1% BSA, and then for 1 hour in TBS +1% BSA + relevant primary antibody. Cells were gently washed twice with TBS, blocked again in TBS +1% BSA for 15 min, and then with TBS +1% BSA + the Alexa 488-labeled relevant secondary antibody for 60 min. Cells were washed again twice with TBS. Fluorescence emission at 525 nm was acquired to determine plasma membrane labeling.

### Biotin-streptavidin Cell Surface Assay

HEK 293 expressing a receptor pair and DRiP78 constructs were washed with PBS and incubated with 0.9 mM EZ-link Sulfo-NHS-LC-Biotin for 30 min. Cells were then washed with PBS (1×) +100 mM Glycine. Samples were then lysed with RIPA buffer. The supernatant was then incubated overnight with Streptavidin Agarose Resin. Samples were washed three times with RIPA buffer and then beads were incubated in SDS-PAGE sample buffer containing 2.8 M DTT for 1 h at room temperature to elute bound proteins. Immunoblots were probed with anti-GFP antibody (1∶1000) and horseradish peroxidase-conjugated secondary antibody (anti-rabbit, 1∶10,000).

### Cell Migration Assay

Jurkat cells transfected with chemokine receptors and DRiP78 constructs were serum starved for 24 h post-transfection. 48 h post-transfection cells were harvested and diluted to 1.5×10^6^ cells/mL using RPMI 1640+0.1% BSA. Cell migration was assayed using a 24 well transwell permeable support system with polycarbonate membrane, pore size 5.0 µm (Costar, Corning, NY). 600 µL of RPMI 1640+0.1%BSA, in presence or absence of 10 ng/mL RANTES, were added to the bottom chamber. 1.5×10^5^ cells were added to the top chamber. Cells were allowed to migrate for 5 h at 37°C and 5% CO_2_. Membrane inserts were then washed once with PBS(1×), fixed using ice cold methanol for 15 min and incubated in a 0.5% Crystal violet solution for 5 min. Membranes were washed with H_2_0 until runoff was clear and air-dried before mounting on a glass slide. At least 3 areas of each membrane observed under 10× objective of an Olympus IX81 equipped with a photometrics coolSNAP HQ2 camera and MetaMorph software was used to quantify cell migration, and cell counts for each field of view were computed using ImageJ software.
